# Brief Research Report: A Pilot Study of Cognitive Behavioral Regulation Therapy (CBT-REG) for Young People at High Risk of Early Transition to Bipolar Disorders

**DOI:** 10.3389/fpsyt.2020.616829

**Published:** 2021-01-22

**Authors:** Jan Scott, Thomas D. Meyer

**Affiliations:** ^1^Institute of Neuroscience, Newcastle University, Newcastle upon Tyne, United Kingdom; ^2^Department of Psychiatry and Behavioral Sciences, McGovern Medical School, University of Texas HSC, Houston, TX, United States

**Keywords:** psychological interventions, early stage, bipolar disorders, adolescent, at risk

## Abstract

Attempts to increase early identification of individuals in the early stages of bipolar disorders (i.e., individuals at high risk of bipolar disorders and/or experiencing a subthreshold syndrome with bipolar symptoms) have highlighted the need to develop high benefit-low risk interventions. We suggest that any new psychological therapy should (i) be acceptable to young people seeking help for the first time, (ii) be applicable to “at risk” conditions and sub-syndromal states and (iii) consider pluripotent factors that may be linked to illness progression not only for bipolar disorders specifically but also for other potential disease trajectories. However, evidence indicates that current interventions for youth with emerging mood disorders mainly represent approaches abbreviated from “disorder-specific” therapies used with older adults and are primarily offered to first episode cases of bipolar disorders who are also receiving psychotropic medication. This brief report discusses empirical findings used to construct core targets for therapeutic interventions that might reduce or delay transition to full-threshold bipolar disorders. We describe an intervention that includes strategies for problem-solving, reducing sleep-wake cycle disturbances, self-management of rumination and that addresses the needs of individuals with “sub-threshold” presentations who are probably at risk of developing a bipolar or other major mental disorders. Outcome data from a case series of 14 youth indicates that the intervention appears to demonstrate a relatively high benefit-to-risk ratio, promising levels of engagement with the therapy modules, and the therapy appears to be acceptable to a wide range of help-seeking youth with early expressions of bipolar psychopathology.

## Introduction

In young people aged <25 years, three of the four most burdensome health problems worldwide are depression, schizophrenia, and bipolar disorders (BD) (1). Given this scenario, early intervention strategies are increasingly advocated for help-seeking adolescents and young adults. To date, most interventions have been “disorder-specific” aiming to reduce or delay onsets of full-threshold episodes of a particular diagnosis. However, it is debatable whether a pure “disorder-specific” strategy is the best option as longitudinal and concurrent comorbidity among psychotic and mood disorders is the rule rather than the exception in youth (2) and those with onsets by age 20 have the greatest risk of developing other mental disorders over the following 15 years (3, 4). Furthermore, subthreshold psychotic, depressive or bipolar syndromes show both homotypic continuity (continuity to the full-threshold disorder most similar to the subthreshold condition) and heterotypic continuity (transition to a different full-threshold disorder) (5).

The complexity and heterogeneity of the evolution of mental disorders presenting during adolescence and early adulthood (i.e., the peak age range for onset of adult-pattern illnesses) has exposed significant concerns regarding the reliability, validity and of traditional diagnoses and the applicability of “disorder-specific” interventions (6). Many experts suggest employing trans-diagnostic models as a more constructive approach for research, prevention, and clinical treatments (6, 7). Whilst this view may not be so critical to individuals with a clearly defined full-threshold first episode or with an established mental disorder, there is increasing support for this approach for individuals deemed “at high risk” of developing major mental disorders in the near future (so-called early transition). The notion of targeting earlier expressions of psychopathology or subthreshold conditions is compatible with the philosophy of clinical staging, which suggests that the evolution of severe mental disorders parallels that of chronic medical disorders (such as cancer, diabetes, etc.), and as such warrant similar approaches to clinical management (6, 7).

Clinical staging models attempt to identify where an individual is located on a “disease continuum” from an asymptomatic state in an individual with enhanced vulnerability for a specific disorder (stage 0) through to end stage disease (stage 4). The application of staging in general medicine has led to the development of interventions for individuals at the earliest clinical stages (usually designated as stages 0 and 1) with the aim of preventing progression to the later stages of illness. For example, risk factors for ischaemic heart disease, that contribute to the early stage presentations (family history of heart disease; obesity; sedentary lifestyle; high cholesterol; borderline increase in blood pressure) are often treated with behavioral or non-pharmacological interventions, with the gradual introduction of stage-specific medications and more complex treatments when the condition become more pronounced (hypertension, angina, etc.).

In psychiatry, the term “early stages” is usually used to describe asymptomatic but at-risk states (stage 0), with early expressions of psychopathology (e.g., mixed presentations with symptoms such as anxiety and depression, etc.) and more discrete subthreshold conditions (stage 1) preceding the first full threshold episode of a disorder (stage 2) (6, 7). For example, in BD, stage 0 can be represented by asymptomatic offspring of a parent with BD (OSBD). Stage 1 cases can be recognized by e.g., the presence of distress or non-specific symptoms in OSBD, or persistent sub-threshold manic symptoms, etc (6, 8). Individuals in stage 1 do not always seek help for their symptoms, but they may come to the attention of clinical services because of distress and/or reduced functioning (8).

The application of staging models to BD is still in its infancy, but the use of “staging frameworks” has led to discussions about the types of treatment modalities that can be targeted at young people in stage 1, i.e., interventions for individuals who do not have a full-threshold mental disorder and traditionally have been excluded from mental health services (8). This is an interesting problem, as it is inappropriate to simply prescribe medications that are used for stage 2 onwards because (a) there is no evidence that the medications used for later stage BD will be helpful in the early stages and (b) only about 20–30% individuals with a condition that meets criteria for Stage 1 will ultimately develop BD (i.e., the disorder for which the medication is recommended) (8). Furthermore, whilst progression to BD is a common transition pathway, the frequency of heterotypic continuity and/or of comorbid psychotic, substance misuse and other disorders has the potential to confound the choice of psychological therapy. So far, this issue is not been considered in detail in the interventions applied to stage 2 BD (i.e., first full-threshold episodes), and approaches for stage 0–1 do not appear to have addressed this problem (8, 9). The therapies for early stage BD described in the literature are potentially low-risk/high benefit, but most are abbreviated, age-appropriate versions of existing BD-specific interventions, and some add peer-groups or family meetings (8, 9). Reviews highlight that these interventions appear to target key issues in BD (such as mood instability) and that family psychoeducation (PED) may be helpful in those who are already receiving treatment (8, 9). However, outcomes of published clinical trials are inconsistent, with lack of evidence of additional benefits compared with support or control interventions (10, 11). Given these findings, and awareness that formal family PED may be unfeasible or unwanted by some adolescents and young adults, we decided to pilot an intervention that could be delivered to individuals, with an option of family sessions if appropriate. We tried to target risk factors for transition to the first full-threshold episode of BD, whilst considering the potential for heterotypic transition and aiming to improve social functioning and/or reduce other sub-threshold problems in those who did not show transition to stage 2. To do this, we examined pluripotent risk factors that may be (a) more prominent in adolescents such as changes in sleep-wake cycle regulation and cognitive-emotional regulation, (b) show associations to impaired functioning and/or the evolution of a range of adult-pattern mental disorders, and (c) may especially be linked to recurrent episodes of mood disorder and/or the onset of BD.

In this brief research article, we provide preliminary data from a pilot study that examined feasibility and acceptability of the therapy (which we describe as CBT-REG i.e., cognitive behavior therapy- regulation model), changes in presenting problems and symptoms, and estimates of changes in core target variables (such as mood, sleep, rumination). Also, we extracted information from clinical records to give an indication of individual outcomes up to 3 years post-intervention where available.

## Methods

We briefly begin by describing the intervention then briefly outline the study protocol (further information is provided in Appendix 1).

### Intervention

We developed a new therapy model, called CBT-REG (cognitive behavior therapy- regulation model) which particularly considered the role of developmental trajectories, comorbidities, and heterotypic outcomes [for details see (12) and (13)]. While first targeting whatever problems led the individual to seeking help, our intervention then focuses on engaging the person into how to manage risk for a mood/mental disorder, alongside potential triggers for mood, activity or sleep variations that occur in the age group. Some sessions specifically focus on two robust developmental characteristics, namely disturbed sleep-wake cycle, and ruminative thinking style. To date, no interventions for young adults have been adapted explicitly to target these mechanisms simultaneously. Given their central importance to CBT-REG and the fact that they may represent underlying pathophysiological mechanisms, we briefly describe these phenomena in Table 1.

**Table 1:** Putative target mechanisms: circadian and cognitive-emotional regulation.

(a) → Target Symptom: Sleep; Target Mechanism: Rest-Activity (Circadian) Regulation Prolonged sleep onset latency and delayed sleep phase both peak in young adults (about 14% show these patterns) and both phenomena show inverse associations with mood and cognitive functioning in non-depressed samples. Further, the degree of circadian disturbance is significantly more marked in those with emerging mood disorders, with >30% young adults with depression showing sleep phase delay according to objective actigraphy recordings. These rest-activity disturbances are reported in about 60% of those with emerging BD. Given the overlap between developmental and disease processes, we postulate that age-recognized shifts in rest-activity rhythms (usually a consequence of circadian dysregulation) act to precipitate or perpetuate illness in individuals at risk of BD or recurrent mood disorders and that targeting these abnormalities can improve outcomes.
(b) → Target symptom: Rumination; Target Mechanism: Cognitive-Emotional Regulation Rumination is defined as a response to negative affect that involves “*repetitively and passively focusing on symptoms of distress and on the possible causes and consequences of the symptoms*.” Rumination may comprise of two elements: an adaptive, reflective-distancing component (akin to “mindfulness”) and a less adaptive, more toxic element referred to as “brooding,” described as “getting depressed about being depressed.” It is a critical marker of cognitive-emotional dysregulation as the individuals' focus on their distress (rather than on distraction to reduce dysphoria) and their passivity (rather than active problem-solving to resolve stressors) act together to intensify their negative affect. Ruminative response style is a robust risk factor for the development and maintenance of psychopathology, especially depression and recent evidence also implicates rumination in anxiety or comorbid anxiety-depressive states in younger (but not older) adults and in BD. Interestingly, those with high levels of rumination also show poorer sleep quality, and abnormal cortisol response to stress. Developmentally, the propensity to rumination peaks during middle and late adolescence (partly because of greater self-focus, etc.), and is more common in females.

**Figure 1:**
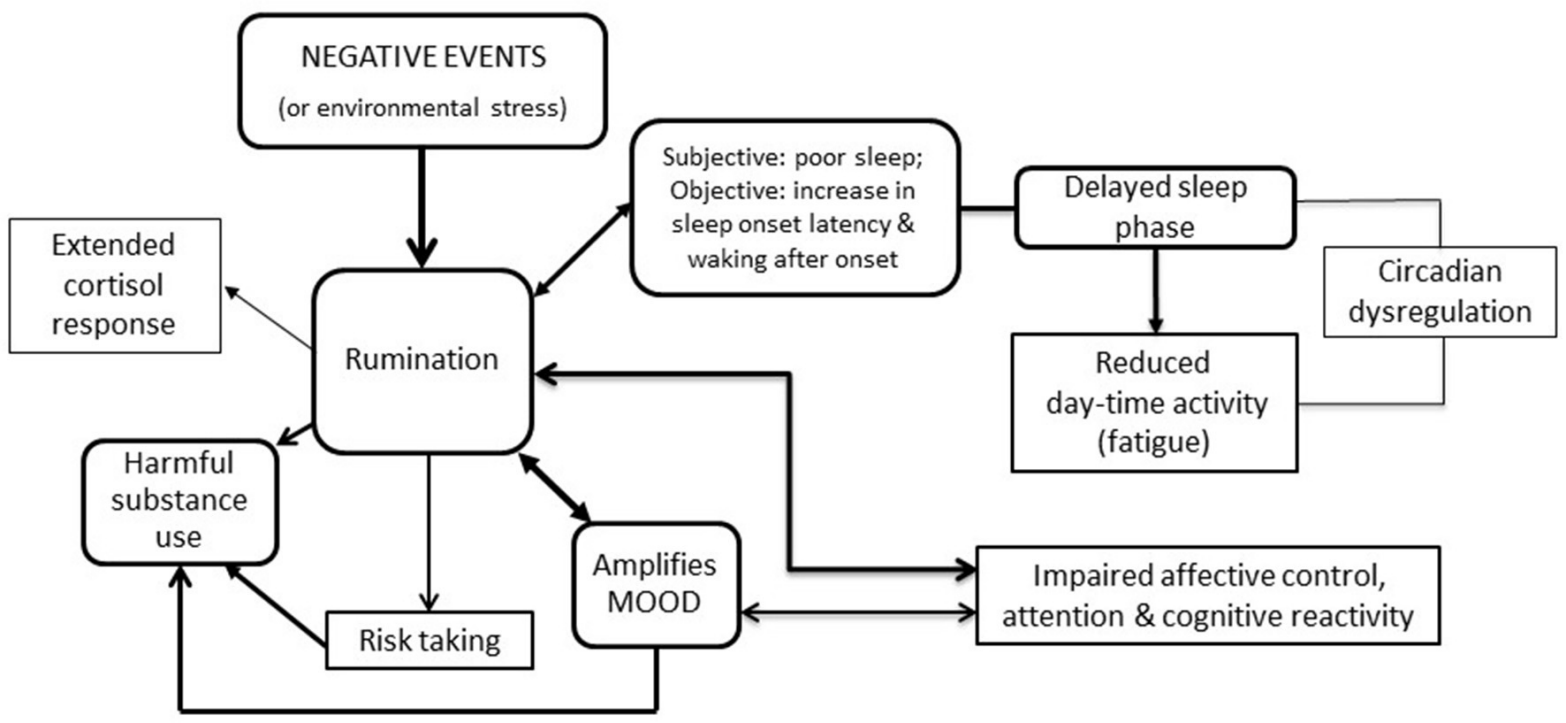
Hypothesized links between rumination, risk taking, mood, substance use, sleep and activity (each link has been demonstrated empirically, but this working model is the first to draw connections between all the different elements).

The rationale for selecting sleep-wake cycle (circadian regulation) and cognitive-emotional processes is because they are known to undergo predictable, developmental changes (in association with frontal lobe maturation) during adolescence and early adulthood (14). Furthermore, there is consistent evidence of inter-relationships between rumination, sleep, behavior, cognition, and mood (see Figure 1). Our model hypothesizes that dysregulation of these rest-activity patterns and cognitive-emotional developmental processes will exacerbate or perpetuate current mood symptoms and increase the risk of early transition from stage 1 to stage 2 or will be associated with persistent academic and social impairments and/or the evolution of other psychopathology (e.g., rumination is also increased in individuals with substance misuse) (14, 15).

The therapy comprises of four modules delivered over about 24 sessions. There is some flexibility in the course, as the duration of the first module (problem-solving and engagement) depends on the nature of the presenting problems and how readily the client engages with therapy. Also, this module offers the possibility of some family sessions if the individuals wants this option (8). The next two modules are approximately eight sessions each. The final module offers practice, skills enhancement etc. The first half of therapy is usually delivered weekly, then the second half is usually every 2 weeks. Although CBT-REG draws on some of traditional elements of Beckian CBT, the focus of CBT-REG interventions is shifted to the examination of thinking processes rather than thought content (as cognitive processing style may be a marker of risk for psychopathology) and to stabilization of the sleep-wake and physical activity-rest cycles (as opposed to daily activity planning).

The second module employs “behavioral regulation” (a modified version of behavioral activation that emphasizes the importance of preventing over-stimulation or excessive activation in those at risk of hypo/mania), alongside sessions targeting sleep-wake cycle or circadian regulation (with techniques derived from CBT for insomnia) (16, 17). Which interventions are initiated is determined by whether sleep disturbances are characterized by insomnia (which is often linked to arousal or anxiety), hypersomnia, unstable patterns, or prolonged sleep onset latency with late waking time (which may be a marker of circadian dysrhythmia) (17–20). Different modules or combined modules are used to regulate these sleep disturbances, although similar daytime interventions are used to manage physical activity across all cases (17, 21).

This next module uses techniques from rumination focused CBT (RfCBT) (22). Although this model has overlapping elements to mindfulness, we use RfCBT with youth for several reasons. For instance, it includes functional analysis and more behavioral elements, linking it well with the second module. Further, it is easier for a wide range of young people to work with RfCBT techniques, as it offers a more concrete approach with less reliance on some of the more subtle mindfulness skills. Additionally, this trans-diagnostic approach is helpful for rumination associated with distress, substance use, anxiety or depressive symptoms, and can also be used to deal with “positive repetitive self-focused thinking” (positive rumination or “basking”) that has been reported in youth with cyclothymia, brief hypomania or other bipolar at-risk syndromes (20).

The RfCBT techniques and the activity-behavioral regulation techniques can also help tackle risk taking behaviors, which are sometimes employed as a maladaptive coping strategy to overcome negative rumination. These modules can be useful in helping to manage potentially harmful substance use and/or risky behaviors associated with sexual activity, etc.

The final sessions (about 4) can be extended over longer time periods if required and focus on recapping the skills and techniques that have been learnt and developing skills in identifying and managing early warning signs (primarily events, triggers, sleep and behavior change) that may indicate risk for increases in symptom levels or reductions in functioning.

The therapy is longer than many interventions for individuals at risk of BD. However, we suggest that this is helpful because it can take 6 months to produce a robust and sustained change in behavior patterns, and cognitive-emotional and sleep-circadian regulation. In addition, young people may prefer to take breaks or have pauses between modules, sometimes to take more time to practice skills from the recent module, but also because of ambivalence about the need to continue to attend sessions (which may be linked with “avoidance” or difficulty in accepting their increased “risk” status). Therapists need to be flexible in their approach, ideally allowing such pauses without formally discharging individuals from therapy, as this avoids difficulties in accessing sessions promptly (especially if symptoms or problems have worsened during any break).

### Participants and Procedure

Participants in this open case series were drawn from individuals recruited to a cohort study entitled “Early identification and treatment of young people at high risk of recurrent mood disorders: a feasibility study.” The cohort study and the pilot study of CBT-REG received ethical approval from the North East of England Research and Ethics committee (Refs: 11/NE/0271 and 12/NE/0325).

The pilot study sought to recruit a minimum of 10 and a maximum of 15 youth aged 16–25 years. Inclusion criteria were: (i) Capable of providing written informed consent (with additional parental consent for those age <18), (ii) Presented in the past 2 years for any problems that are/were considered to be mood-related according to a clinician working in primary care or secondary health services (such as GP clinics and/or Child and Adolescent Mental Health Services, Youth Drug and Alcohol services, adult psychiatry, crisis assessment and treatment, and/or Early Intervention in Psychosis services), and (iii) Currently help-seeking and identified as being “at risk” of BD (i.e., they met criteria for stage 0 or 1 for BD). The latter was ascertained by a comprehensive interview that included a structured clinical interview for Axis I and II diagnoses, a detailed assessment of family history, instruments used to screen for BD (e.g., General Behavior Inventory) (23), etc.

Exclusion Criteria were: (i) Evidence of the current or lifetime presence of a Bipolar I or Bipolar II Disorder diagnosed according to internationally recognized criteria (i.e., they already met criteria for stage 2 for BD), (ii) Currently being prescribed a mood stabilizer or long-term treatment with an atypical antipsychotic (iii) Clinical diagnosis of severe Borderline or Antisocial Personality disorder, and/or clinical high risk of deliberate self-harm or suicidal behaviors, (iv) Insufficient knowledge of English language, and/or (v) Other characteristics that were likely to significantly impair their ability to participate in a verbal therapy.

Individuals who met eligibility criteria, gave written informed consent, and completed the baseline assessment procedure were offered the opportunity to commence therapy.

### Measures

Given the exploratory nature of this study, we included many different measurement scales (and some participants also used electronic monitoring) and findings from some of the observer, subjective and objective ratings have been reported elsewhere (8, 17, 24, 25). Appendix 1 details the assessments, to summarize, we recorded-

1. Socio-demographics and clinical characteristics
2. Pre-and-post-therapy self-ratings of the 90-item Symptom Checklist (SCL-90R) (26), the Internal State Scale (ISS) (27) and the Work and Social Adjustment scale (WASA) (28). The ISS provided the main measure of symptoms over time (27). This self-rating comprises 16 items (each rated on 0–100 Likert scale) and allows simultaneous recording of manic, depressive, and psychotic symptoms. The 16 items are divided into four subscales: Activation (ISS-ACT); Depression (ISS-DEP); Perceived Conflict (a measure of psychotic symptoms: ISS-PC); and Well-Being (ISS-WB).
  To explore therapy targets we used the Ruminative Response Scale of the Response Styles Questionnaire (RSQ) (29) and extracted data for four key metrics selected from a self-rated sleep diary, namely: bedtime (BT), sleep onset latency (SOL), total sleep time (TST) and rise time (RT) for two consecutive weekends before and two consecutive weekends at the end of therapy. We report weekends as these represent un-entrained sleep patterns, which are likely to be better markers of sleep-wake cycle problems than weekdays (where routines are imposed by external factors such as educational class times) (20, 30).
3. Post-therapy we assessed acceptability of therapy by examining number of sessions attended and the number of dropouts. Also, we asked participants for feedback about CBT-REG regarding usefulness and difficulty of modules, satisfaction with therapy (rated 0-10) and whether would they recommend CBT-REG to others.
4. Course and Outcome: We examined clinical records for about 2 years post-therapy.

### Data Analysis

We report means and standard deviations (SD) or medians and interquartile ranges (IQR) for continuous measures and counts or percentages for categorical measures. We estimated response to CBT-REG using within-group effect sizes (ES with 95% confidence intervals); large ES were defined as Cohen's *d* ≥ 0.80, with medium ES defined as Cohen's *d* ≥ 0.40 (up to 0.79).

## Results

The case series comprised 8 females and 6 males with a median age of 18.5 years (IQR-16.3–20.7), 50% (*n* = 7) resided with one or both parents; most (*n* = 9) were in late secondary or undergraduate tertiary education, two were in training, two were in paid employment and one was currently unemployed. Three individuals had a family history of BD with or without alcohol/substance misuse and two had a family history of major depression. Four individuals were seeking help for depressive and anxiety symptoms, five for intermittent or ongoing subthreshold hypomanic symptoms (with or without depression), two reported educational problems associated with cannabis and/or alcohol use, and the others reported less specific symptoms but identified a range of distressing problems such as social impairment, academic difficulties and sleep disturbances that impacted on functioning. Four individuals were receiving antidepressants for depressive or anxiety symptoms.

As shown in Table 2, large ES were observed for change in ISS-DEP, ISS-ACT, SCL, and RSQ (with positive 95% confidence intervals). Moderate ES were noted for ISS-PC and WASA, but 95% confidence intervals indicate these ES are less robust. It was notable that sleep metrics showed lower ES, with only RT demonstrating a medium ES (*d* = 0.51). The 95% confidence intervals indicate the need for careful interpretation, but the finding is noteworthy as RT is a core target for CBT-I and is especially linked to re-setting sleep-wake cycles. Also, it should be noted that reporting only mean BT, SOL, TST metrics over time may be misleading in youth as some individuals will report insomnia whilst others may report hypersomnia, etc. As such, the goal of therapy was to increase the TST (and/or shorten SOL) for the former group and to shorten TST (and modify SOL and RT) for the latter.

**Table 2:** Scores pre- and post-CBT-R for 14 participants who commenced therapy.

**Measure**	**Pre-therapy Mean (SD)**	**Post-therapy Mean (SD)**	**ES**^****a****^ **and 95% CI**	
**Internal state scale**			**ES**	**95% CI**
Depression	83.21 (69.87)	34.37 (26.57)	*0.92*	*0.12, 1.67*
Well-being^*^	108.97 (52.14)	130.14 (51.32)	0.41	−0.35, 1.15
Activation	104.73 (63.21)	62.98 (37.29)	*0.80*	*0.00, 1.55*
Perceived conflict	76.45 (67.22)	47.58 (32.91)	0.54	−0.21, 1.27
**Symptom checklist**	239.27 (53.39)	187.22 (50.42)	*1.00*	*0.02, 1.76*
Work and social adjustment^*^	9.91 (6.82)	14.70 (8.47)	0.63	−0.12, 1.36
**Response styles questionnaire**	57.81 (8.96)	42.33 (12.78)	*1.40*	*0.54, 2.18*
**Sleep diary metrics**				
Bedtime: 24 h clock (SD in hours)	00.35 (2.10)	23.67 (1.58)	0.37	−0.30, 1.13
Sleep onset latency in minutes	42.80 (34.71)	31.63 (22.47)	0.38	−0.31, 1.22
Total sleep time in minutes	417.00 (92.62)	425.45 (71.20)	0.10	−0.64, .86
Rise time: 24 h clock (SD in hours)	09:31 (1.65)	08:47 (1.24)	0.51	−0.02, 1.31

* *Higher score for these variables indicates a better outcome (for all other self-report variables, lower scores indicate better outcomes)*.

a *ES, Effect Size, estimated as Cohen's d*.

*95% CI: 95% confidence intervals for the ES*.

*Data shown in italics indicate significant findings*.

Acceptability was partly inferred from the CBT-REG completion rate. One individual attended only four initial sessions and dropped out after resolving some initial problems (interestingly, we discovered that they later returned to therapy). Two individuals dropped out after completing 14 sessions (i.e., they terminated therapy during sessions addressing sleep-wake cycle disturbances and development of preventative strategies). So, the attrition rate was 21% (lower than many studies of CBT or of psychological therapies in adults with clearly established diagnoses). The average number of sessions for CBT-REG completers was 22. Three individuals also received 2-3 family sessions. Twelve individuals provided feedback, the mean satisfaction score was of 8.40 (SD 1.95) and eight individuals stated they would and two probably would recommend the intervention to others. Two males reported some difficulties in undertaking rumination-focused interventions independently, and six individuals indicated they needed more time to implement all the different sleep-wake cycle interventions alongside developing long-term self-monitoring/self-management plans.

Follow-up revealed that two participants (one of whom had dropped out of therapy) had a full-threshold BD or psychotic disorder. One other youth, originally referred with depression and a family history of BD, was reported to have experienced a further depressive episode and had subthreshold hypomanic syndromes (but had not developed BD so far). Six individuals had been discharged from secondary care services and there was no evidence of any further significant mental health problems.

## Conclusions

We are mindful that this is a small proof of principle study and that replication is required in larger-scale double blind randomized controlled trials in which the CBT-REG model described is compared with at least one other control condition. However, we are encouraged by the findings of this pilot study. Of course, the use of a case series design, reliance on self-report data to assess outcomes, and lack of adjustment of analyses for potential confounders (such as age, sex, clinical presentations, etc.) means the magnitude of the reported ES may be inflated, and the 95% confidence intervals serve as a reminder to interpret even moderately large ES carefully. Nevertheless, it is reassuring that large changes occurred in a broad range of distressing symptoms (as measured by the SCL) and in specific key targets namely depression and activation (as measured by the ISS) and rumination. Also, changes in social and behavioral regulation (as measured by the WASA) and RT were moderately large, albeit with varying confidence intervals. Given the robust data supporting the use of CBT in a wide range of major mental disorders and across all age groups, it is unsurprising that SCL and depression scores significantly decreased. Further, given the use of key strategies derived from RfCBT, is was anticipated that participants would show significant reductions in rumination. The modification/stabilization of activation (a proxy for manic psychopathology in the ISS) is notable, especially given that there was less evidence for the overall effect of CBT-REG on sleep-wake cycle disturbances. However, it is possible that the benefits of therapy on sleep disturbances may be under-estimated as we only extracted data for a few variables from the sleep diaries and focused only on average weekend measures of those metrics. This alternative explanation of the sleep findings should be born in mind as there is considerable evidence that variability in key sleep parameters may be more sensitive markers of the overall health of the sleep and circadian system, especially in BD (18, 30). Of course, given the feedback from participants that the strategies presented in the later sessions of CBT-REG were more difficult to instigate independently, it may be that more work needs to be undertaken on the balance of sessions within CBT-REG in an attempt to enhance the utility of the selected strategies (derived from CBT-I) in adolescent and young adult populations. Also, it needs to be emphasized that that the key problem(s) for which the individuals sought help was tackled first, meaning that the topics addressed later in therapy require a personal acknowledgment of a potentially increased risk for mental health problems (31).

Although case note follow-up only provides weak evidence, we do think it is worthwhile to get a snapshot of the course and outcome of case series participants. Recent research highlights that about 18% individuals with a family history of BD will develop BD or another major mental disorder during the peak age range for onset of adult pattern conditions (4, 6, 7, 31–34). Furthermore, about 10% of those with early expressions of psychopathology and about 30% of those with subthreshold manifestations of BD will develop a full-threshold mood or psychotic disorder over about 2–4 years (31–33). In those with a family history of BD and a subthreshold presentation of BD, the transition rate may be >50% over 2 years (31–34). In this pilot study of a small case series, we found evidence that about 21% could be viewed as having a poor clinical outcome with 7% of participants (one individual) developing BD, 7% a psychotic disorder, and 7% continuing to experience mood problems, but without transition to full-threshold BD (or perhaps transition has been delayed). It is recognized that individuals who do not show transition from subthreshold to full-threshold disorders are not necessarily well, and many continue to experience a range of clinical and social impairments (4, 6, 7). As such, it is encouraging that 43% of the case series (six individuals) were known to be well enough to be fully discharged from mental health care.

A review of current interventions for individuals with emerging BD identifies that the available treatment protocols include the necessary elements to treat full-threshold BD and that the therapies give due consideration to maturational level or social context (eg. specific sessions focused on the young person's functioning at school, managing peer group pressures, individuation from parents, etc.) (8, 9). However, whilst this makes the therapies useful for young people at stage 2 of BD (i.e., already meeting criteria for a full-threshold syndrome) it is unclear if they provide sufficient strategies to comprehensively target individual needs or any unique prognostic indicators (12, 13). Also, we note that whilst family therapy is particularly efficacious in young people with emerging BD (8, 9) many of the individuals recruited to our study were already living independently (and some were residing at a long distance form their family) and only half the remaining individuals opted to engage in family sessions. This suggests that peer group or individual therapy will be an important option for many older adolescents and young adults. Also, few of the available therapies target underlying trans-diagnostic factors such as rumination (which may be associated with co-morbid substance misuse and other problems such as over-arousal, irritability and anxiety, etc.).

As early identification of individuals at risk of bipolar and other recurrent mood disorders is now being viewed as an important unmet need, we are likely to increase the service use by these groups (in the same was as was seen with early intervention in psychosis). A significant challenge is to develop more valid, evidence-based, timely interventions, that are appropriate for the early stages of BD and consider the reality of the trans-diagnostic outcomes seen in these groups of help-seeking adolescents and young adults. This requires considerable efforts from researchers in psychological therapies and potentially even greater rethinking of the underlying mechanisms that needed to be targeted by novel pharmacotherapies. We believe that our pilot study is a useful first step in trying to address the complex issues faced in trying to deliver a psychological intervention that might prevent transition to a specific disorder whilst addressing the pluri-potentiality of risk in this age and stage group, and also acknowledges the need to tackle any presenting problems or conditions that led the individual to seek clinical input in the first place. A key message from our work to date is that it is likely that the therapy for youth at risk of BD may need to be longer than anticipated, which means it is also worthwhile considering the optimal format for delivery (e.g., intermittent models with pauses, or a set of core modules with booster sessions, etc.).

## Data Availability Statement

The data analyzed in this study is subject to the following licenses/restrictions: The Ethics Committee and study participants only gave permission to the EIMD researchers to use their data. Requests to access these datasets should be directed to jan.scott@newcastle.ac.uk.

## Ethics Statement

The studies involving human participants were reviewed and approved by The Cohort Study and the Pilot Study of CBT-REG received ethical approval from the North East of England Research and Ethics Committee (Refs: 11/NE/0271 and 12/NE/0325). Written informed consent to participate in this study was provided by the participants' legal guardian/next of kin.

## Author Contributions

JS and TM were CI and senior PI respectively on the Early identification and treatment of young people at high risk of recurrent mood disorders: a feasibility study. They jointly supervised the research team, undertook the main study and the pilot study. JS wrote the preliminary draft of this report and TM undertook redrafting, both have approved the final manuscript. All authors contributed to the article and approved the submitted version.

## Conflict of Interest

The authors declare that the research was conducted in the absence of any commercial or financial relationships that could be construed as a potential conflict of interest.
