# Supplementary material for: Brief Research Report: A Pilot Study of Cognitive Behavioral Regulation Therapy (CBT-REG) for Young People at High Risk of Early Transition to Bipolar Disorders
Source: Front Psychiatry. 2021 Jan 22;11:616829. doi: 10.3389/fpsyt.2020.616829 (PMC7874073; doi:10.3389/fpsyt.2020.616829)
Supplement: Supplementary file 1 [file Data_Sheet_1.docx]

**Appendix 1**

Further details of the pilot study protocol.

**Participants and Procedure**

Participants in this open case series were drawn from individuals recruited to a cohort study entitled ‘Early identification and treatment of young people at high risk of recurrent mood disorders: a feasibility study’. The cohort study comprised a planned prospective follow-up of about 150 young people over 12-36-month (and is described in detail elsewhere). The cohort study and the pilot study of CBT-REG received ethical approval from the North East of England Research and Ethics committee (Refs: 11/NE/0271 and 12/NE/0325). Ethical approval for the CBT-REG study was obtained during the final year of the recruitment phase of the cohort study.

The pilot study sought to recruit a minimum of 10 and a maximum of 15 youth aged 16-25 years. Inclusion criteria were: (i) Capable of providing written informed consent (with additional parental consent for those age<18), (ii) Presented in the past 2 years for any problems that are/were considered to be mood-related according to a clinician working in primary care or secondary health services (such as GP clinics and/or Child and Adolescent Mental Health Services, Youth Drug and Alcohol services, adult psychiatry, crisis assessment and treatment, and/or Early Intervention in Psychosis services), and (iii) Currently help-seeking and identified as being ‘at risk’ of BD (i.e. they met criteria for stage 0 or 1 for BD). The latter was ascertained by a comprehensive interview that included a structured clinical interview for Axis I and II diagnoses, a detailed assessment of family history, instruments used to screen for BD (e.g. General Behaviour Inventory) (23), etc.

Exclusion Criteria were: (i) Evidence of the current or lifetime presence of a Bipolar I or Bipolar II Disorder diagnosed according to internationally recognized criteria (i.e. they already met criteria for stage 2 for BD), (ii) Currently being prescribed a mood stabilizer or long-term treatment with an atypical antipsychotic (iii) Clinical diagnosis of severe Borderline or Antisocial Personality disorder, and/or clinical high risk of deliberate self-harm or suicidal behaviours, (iv) Insufficient knowledge of English language, and/or (v) Other characteristics that were likely to significantly impair their ability to participate in a verbal therapy.

Individuals who met eligibility criteria, gave written informed consent, and completed the baseline assessment procedure were offered the opportunity to commence therapy.

**Measures**

Given the exploratory nature of this study, we included many different measurement scales (and some participants also used electronic monitoring) and findings from some of the observer, subjective and objective ratings have been reported elsewhere (8, 17, 24, 25). Here, we focus on unpublished findings for ratings of established and robust measures that have been widely used in other therapy outcome studies.

1. Socio-demographics and clinical characteristics: key demographic and clinical variables were recorded.
2. Pre-and-post-therapy self-ratings:

(a) Symptoms and Functioning-

General Symptoms and Problems: To capture information on a broad range of symptoms we used the revised versions of the 90-item Symptom Checklist (SCL-90R). Each item is assessed on a 100-mm, anchored scale and covers multiple dimensions ranging from somatic complaints, obsessive-compulsive patterns, interpersonal sensitivity, anxiety, depression, hostility, phobic behavior, paranoid ideation, and emotional alienation. Previous studies suggest that these ratings are reliable indicators of an individuals’ emotional functioning (26).

Mood and Psychotic Symptoms: We used the Internal State Scale (ISS) as the main measure of symptoms over time (27). This self-rating comprises 16 items (each rated on 0–100 Likert scale) and allows simultaneous recording of manic, depressive, and psychotic symptoms. The 16 items are divided into four subscales: Activation (ISS-ACT); Depression (ISS-DEP); Perceived Conﬂict (a measure of psychotic symptoms: ISS-PC); and Well-Being (ISS-WB).

Behaviour and Social Functioning: We used the Work and Social Adjustment scale (WASA) to capture regularity of behaviour patterns and satisfaction with work (or education and training) and social functioning (28). This brief questionnaire assesses ﬁve aspects of day-today life, namely: work/education engagement, individual social activities, social activities involving others, home life and sex life. Each area is rated on a 0–8 scale, with higher scores indicating more regular patterns and better functioning. Scores on each subscale were combined to give an overall WASA rating (range 0–40).

1. Specific Therapy Targets-

Ruminative Response Style: Rumination was measured using the Ruminative Response Scale of the Response Styles Questionnaire (RSQ) (29). The RSQ comprises 22 items that assess ruminative responses to sad and depressed mood (score range 22–88). Participants rate the frequency that they use ruminative strategies, and higher scores connote higher levels of rumination.

Sleep-Wake Cycle: we extracted data for four key metrics selected from a self-rated sleep diary, namely: bedtime (BT), sleep onset latency (SOL), total sleep time (TST) and rise time (RT) for two consecutive weekends before and two consecutive weekends at the end of therapy. We focus on these items as they capture features of different types of sleep disturbance such as insomnia (SOL and TST), hypersomnia (TST), and misalignment of the sleep-wake cycle (BT, TST, and RT). Also, and very importantly in youth, weekends represent un-entrained sleep patterns, which are likely to be better markers of sleep-wake cycle problems than weekdays (where routines are imposed by external factors such as educational class times) (20, 30).

1. Measures rated post-therapy only-
2. Acceptability: At the end of the course of therapy, we noted the number of sessions attended and the number of dropouts. Also, we asked participants for feedback about CBT-REG regarding four themes: which therapy module(s) did they find most useful personally; which module(s) did they find most difficult to understand or utilize; personal satisfaction with therapy (rated 0-10, with high scores indicating greater satisfaction); and whether would they recommend CBT-REG to others seeking help for ‘similar problems and difficulties’ to their own (rated: no, unsure, probably, yes). Individuals were also asked open-ended questions regarding therapy and the assessments, etc. (qualitative analysis of these items is not reported here).
3. Course & Outcome: We examined clinical records to identify whether individuals developed BD in the two years post-therapy, whether they continued to have mental health problems and the nature of any ongoing conditions.

**Data Analysis**

Descriptive data are reported as means and standard deviations (SD) or medians and interquartile ranges (IQR) for continuous measures and counts or percentages for categorical measures.

Data for the SCL, ISS, WASA, and RSQ were collected immediately prior to commencing therapy, at about 12-14 weeks (approximate mid-point) and at the end of therapy. Sleep diary metrics (along with a mood diary) was collected throughout therapy, but as noted above we use only selected recordings from the beginning and end of therapy (to maintain comparability with the other cross-sectional measures). In this article, we estimated response to CBT-R by comparing measures as rated at the pre- and post-therapy assessment points. Given the small sample we employed last observation carried forward to replace the sporadic individual missing data. Within-group effect sizes (ES with 95% confidence intervals) were calculated (Cohen's d); large ES were defined as d⩾0.80, with medium ES defined as d⩾0.40 (up to 0.79).
